# Characterization of immune responses of human PBMCs infected with *Mycobacterium tuberculosis* H37Ra: Impact of donor declared BCG vaccination history on immune responses and *M*. *tuberculosis* growth

**DOI:** 10.1371/journal.pone.0203822

**Published:** 2018-09-11

**Authors:** Sudha Bhavanam, Gina R. Rayat, Monika Keelan, Dennis Kunimoto, Steven J. Drews

**Affiliations:** 1 Department of Laboratory Medicine and Pathology, University of Alberta, Edmonton, Alberta, Canada; 2 Department of Surgery, Ray Rajotte Surgical-Medical Research Institute, Alberta Diabetes Institute, University of Alberta, Edmonton, Alberta, Canada; 3 Department of Medicine, University of Alberta, Edmonton, Alberta, Canada; 4 Provincial Laboratory for Public Health, University of Alberta Hospital, Edmonton, Alberta, Canada; Fundació Institut d’Investigació en Ciències de la Salut Germans Trias i Pujol, Universitat Autònoma de Barcelona, SPAIN

## Abstract

This study characterized the immune responses in early *Mycobacterium tuberculosis* (*Mtb)* H37Ra infection of human peripheral blood mononuclear cell (PBMC)-collagen matrix culture and the impact of Bacille Calmette-Guérin (BCG) vaccination history of donor PBMCs on the immune responses to *Mtb* infection. Aggregates of PBMCs were initially observed on day 3 and the size of aggregates continued to increase on day 8 post-infection, where macrophages and T cell subsets were identified to be present. Similarly, mycobacterial load progressively increased in infected PBMCs during the 8 days of culture but were significantly lower in infected PBMCs from BCG vaccinated (BCG+) donors compared to unvaccinated (BCG-) donors. The levels of INF-γ, TNF-α, IL-4, IL-6, IL-10 and IL-17 in the supernatants of *Mtb*-infected PBMCs peaked at day 3 and decreased on days 5 and 8. The levels of these cytokines except IL-10 were significantly lower in *Mtb*-infected PBMCs from BCG+ donors compared to infected PBMCs from BCG- donors. The percentages of activated naïve Th cells, activated effector memory Th cells and activated central memory Tc cells were significantly higher in *Mtb*-infected PBMCs compared to uninfected PBMCs at day 8 post-infection. Further, the proportion of activated central memory Tc cells was significantly higher in infected PBMCs from BCG+ donors compared to the BCG- donors. This study highlights the possibility that BCG vaccination may confound results that utilize human PBMCs to study *Mtb* infection.

## Introduction

Tuberculosis (TB) infection affects approximately one in three people in the world and causes approximately 1.5 million deaths worldwide each year [1]. The disease is caused by *Mycobacterium tuberculosis* complex (*Mtb*), which is comprised of several human and animal associated species and sub-species [2]. Following infection of the lung, the *Mtb* bacillus is phagocytosed by dendritic cells (DCs) and monocyte-derived macrophages [3–6] where the bacillus survives within these cells [7]. The host cellular immune response to *Mtb* infection includes the recruitment of new macrophages [8–11] and T cells from the circulation to the site of infection within the parenchyma of the lung. These recruited immune cells interact with the pre-existing macrophages and DCs in the lung in support of the immune response against *Mtb* infection [12]. This series of events leads to the formation of a mature granuloma, a multicellular structure composed of infected and uninfected macrophages, epithelioid cells, giant cells (multinucleated cells derived from fused macrophages), T cells and B cells to contain the bacilli and to prevent spread of the *Mtb* infection [13–15].

We have previously reviewed a variety of *in vitro* approaches to better understand the development of a granuloma and to control the pathophysiology of *Mtb* [16]. Due to the limited access to human biopsy samples of granulomas, several three-dimensional *in vitro* models have been used to study the structure and function of granulomas. In particular, the three-dimensional *in vitro* granuloma model of *Mtb* infection which consists of donor PBMCs in a collagen matrix [17] has allowed the study of host factors that drive the formation of a granuloma [15,18,19]. Human PBMCs infected with members of the *Mtb* complex formed aggregates of bacteria and monocyte-derived macrophages as well as T cells, which may represent an early granuloma formation [16,17,19–23].

In this study, we used the three-dimensional *in vitro* granuloma model of *Mtb* infection and characterized the human immune response to attenuated *Mtb* H37Ra. Although use of attenuated strains in infection models may not mirror infection with wild-type virulent strains, use of attenuated strains allow us to assess the impact of potential confounders on experimental models using tools that are outside of a Biosafety Level 3 laboratory [24]. We postulated that one key confounder of infection models that should be monitored in experiments is Bacille Calmette-Guérin (BCG) vaccination history of PBMC donors. Some evidence suggests that a history of BCG vaccination may influence results of studies using *in vitro* granuloma models by generating protection, albeit variable, against *Mtb* infection, and would be a significant confounder of *in vitro* studies [25]. Given the sparse literature in the field, this study was initiated with two aims. The first was to characterize the early host immune responses in human PBMCs infected with an attenuated *Mtb* H37Ra strain, as well as the growth of this strain during infection. The second aim was to determine the impact of BCG vaccination history of PBMC donors on the immune and bacterial responses in three dimensional *in vitro* granuloma model of *Mtb* infection.

## Materials and methods

Materials were obtained from Fisher Scientific, Ottawa, Ontario unless stated otherwise.

### Ethics statement

This study was approved by the University of Alberta Health Research Ethics Board (Pro00057636) and all methods were performed in accordance with institutional guidelines and regulations. Informed written consent was obtained from all study participants.

### PBMC donor enrollment

**Fig 1:**
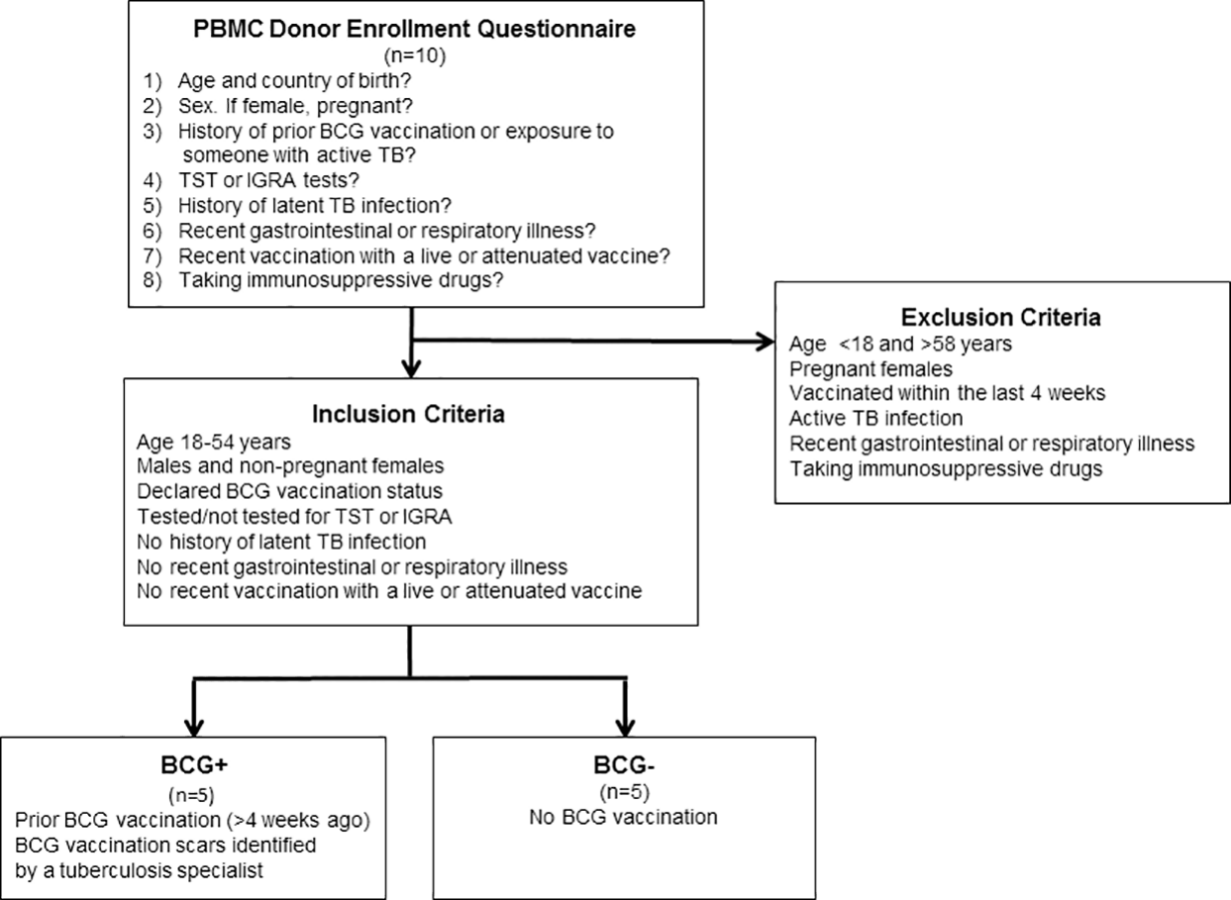
Flow diagram of donor selection criteria.

A questionnaire approved by the institutional ethics board was used to assess potential confounders in blood donors. Potential donors were asked 1) what is their age and country of birth, 2) for female donors, if they were pregnant, 3) if they recalled a prior BCG vaccination or exposure to someone with active TB, 4) if they had previously tested positive with a tuberculin skin test (TST) / interferon-gamma release assay (IGRA), 5) if they had a history of latent TB infection, 6) if they had a recent gastrointestinal or respiratory illness, 7) if they were vaccinated with a live or attenuated vaccine within the previous four weeks, and 8) if they were taking immunosuppressive drugs (Fig 1). In regards to donor TST history, each donor was asked about TST history on the donor history form. This included if the donor had any previous TST test done (“yes” or “no”). If the donor responded “yes” to a history of a TST, then the donor was asked if the TST test was positive, negative or if the donor was not sure about the TST result.

### Donor inclusion criteria

Healthy males and non-pregnant females between the ages of 18–54 years with no history of latent TB infection or recent gastrointestinal or respiratory illness or recent vaccination, and not taking immunosuppressive drugs with or without the potential confounders listed above were included in this study.

### Donor exclusion criteria

Males and females under the age of 18 years, pregnant females, individuals who were vaccinated within the last 4 weeks with live or attenuated vaccine, individuals with active TB infection, individuals with a recent gastrointestinal or respiratory illness, or individuals who were taking any immunosuppressive drugs were excluded from the study. Individuals over age 54 were excluded from the study to avoid any age-related changes that may skew the results.

### Presence of BCG vaccination scars

Volunteer blood donors who declared a BCG vaccination history had both arms inspected for scars consistent with BCG vaccination by DK. a tuberculosis specialist for more than 25 years.

### Isolation of PBMCs from human blood

Volunteer donors gave informed written consent to collect 50 ml blood to isolate and use their PBMCs for research purposes. Blood was collected in vacutainers containing sodium heparin anticoagulant by phlebotomists at the Alberta Diabetes Institute Clinical Research Unit of the University of Alberta. PBMCs were isolated from whole peripheral blood following our standard protocol using density gradient centrifugation. The blood was diluted 1:1 in sterile saline and layered onto Lympholyte^®^-H cell separation media (Cedarlane, Burlington, Ontario) and centrifuged at 870 x g, for 30 min at room temperature (IEC Centrifuge Model CRU-5000, O’Fallon, Missouri) without brakes. The interphase between the upper phase (plasma, thrombocytes) and the lower phase (Lympholyte^®^-H) containing the PBMCs was carefully transferred to a fresh tube using a Pasteur pipette and washed twice with phosphate-buffered saline (PBS). Each wash consisted of resuspending the cells in 50 ml PBS followed by centrifugation at 200 x g, for 5 min at 4°C to pellet the cells and to remove the supernatant (Beckman Coulter Allegra X-15R centrifuge, Mississauga, Ontario). A hemocytometer was used to count the total number of live and dead cells and cell viability by 0.4% Trypan Blue exclusion dye (Sigma-Aldrich, Oakville, Ontario). For cryopreservation, the PBMCs were adjusted to a final concentration of 1.5–2.0 x 10^7^ cells/ml by slowly adding appropriate volumes of ice-cold cryopreservation medium, which consisted of 10% dimethyl sulfoxide (DMSO) (Sigma-Aldrich) and 90% heat-inactivated fetal bovine serum (FBS). One ml aliquots of the cell suspension were transferred into 1.2 ml Nunc cryopreservation vials and stored at -80°C overnight and then in liquid nitrogen (-196°C).

When needed, the vials were removed from liquid nitrogen storage and thawed for 2 min in a 37°C water bath (Innova 3100 Water Bath Shaker, New Brunswick Scientific, Edison, New Jersey). Cells were immediately removed from the vials with a sterile pipette and diluted in 20 ml of fresh culture medium consisting of Roswell Park Memorial Institute medium (RPMI) (Sigma-Aldrich) supplemented with 1% Penicillin-Streptomycin and 10% FBS. The cell suspension was centrifuged for 5 min at 72 x g to allow removal of the cryopreservation medium. The cells were resuspended in RPMI culture medium, 20 μl of DNase solution (Sigma- Aldrich) was added and then incubated for 30 min in a 37°C water bath. The cells were then filtered through Falcon™ Cell Strainers to remove the cell debris, pelleted by centrifugation at 200 x g for 5 min at room temperature.

### Characterization of donor PBMCs

PBMCs were prepared for flow cytometry to characterize different subsets of cell populations (e.g. T cells, B cells, Treg cells, macrophages, dendritic cells (DCs) and Th1, Th2 and Th17). Cell pellets were resuspended in FACS buffer (PBS supplemented with 10% heat-inactivated FBS). The final concentration of each cell suspension was adjusted to 1 x 10^6^ cells/ml in ice-cold FACS buffer and transferred to Falcon™ round bottom polystyrene tubes. Cells were washed two times, where each time cells were resuspended in cold PBS, then centrifuged at 200 x g for 5 min and the supernatant discarded. Cell pellets were resuspended in 100 μl of Human BD Fc block (BD Biosciences, Mississauga, Ontario), diluted 1:50 in FACS buffer and incubated for 30 min on ice to avoid any non-specific binding and background fluorescence. Each panel of cells were stained with a cocktail of antibodies directly conjugated to fluorochromes (S1 Table, BD Biosciences) by adding an appropriate concentration of antibody as per manufacturer’s recommendation and incubated for 30 min on ice in the dark. Cells were then washed 3 times, where each time cells were resuspended in cold PBS, centrifuged at 525 x g for 5 min and the supernatant discarded. The cells were then resuspended in 400 μl of ice-cold FACS buffer and stored at 4°C in the refrigerator until analysis on the same day. Since multiple fluorochromes were used for each panel, compensation tubes (BD Biosciences) were used to distinguish each flourochrome and to avoid any spectral overlap. Compensation beads were stained as described above.

### Flow cytometry analysis and data acquisition

Data was acquired by running the samples on the BD FACSCanto^TM^ II system (BD Biosciences) with 10,000 events collected for each tube and analyzed with the BD FACSDiva^TM^ software (BD Biosciences). First, the cells were gated using the forward scatter (FSC) and side scatter (SSC) to find viable, single cell events. Gating excluded events with low FSC and high SSC. Using a bivariate histogram, four different populations of cells were analyzed: double-positive, single positive for each antibody, and negative for both. The percent of each cell population queried was automatically generated by the software in each quadrant and was compared among the samples.

### Growth of bacterial strains used in the study

An aliquot of *Mtb* H37Ra (ATCC 25177) was obtained from the American Type Culture Collection (ATCC) (Cedarlane). Bacteria were fast-thawed in a 37°C water bath (Innova 3100 Water Bath Shaker). The bacteria were centrifuged at 3000 x g for 20 min and were cultured in 15 ml conical Pyrex tubes containing 5 ml Difco 7H9 broth Middlebrook supplemented with 10% ADC (albumin, dextrose, sodium chloride, catalase) enrichment and 0.05% Tween 80 (BD Biosciences) at 37°C without agitation for 3–4 weeks. Optical density was measured at 600 nm on 1 ml aliquots of bacterial suspension removed from the culture every 3–4 days (Pharmacia Biotech Ultrospec 3000 UV/Visible Spectrophotometer, Scinteck Instruments, Manassas Park, Virginia) until it corresponded to the log phase (OD_600_ = 0.4–0.5) of bacterial growth [26]. Once the bacteria reached log phase, they were harvested by centrifugation for 5 min at 1200 x g and then resuspended in an equal volume of fresh Difco Middlebrook 7H9 medium. Ten-fold serial dilutions of each suspension were plated onto Middlebrook 7H11 Selective Agar (BD Biosciences) and incubated at 37°C with 5% CO_2_ between 21 and 28 days. The bacterial concentration (CFUs/ml) was determined by dividing the number of CFUs by the product of the dilution and the volume of the plated dilution. *Mtb* H37Ra were then centrifuged at 3000 x g for 20 min to pellet the bacteria. The bacteria were resuspended in PBS and cryopreserved in PBS.

*Escherichia coli* (*E*. *coli*) and *Staphylococcus aureus* (*S*. *aureus*) were obtained from the culture collection of the Provincial Laboratory for Public Health, Edmonton, Alberta. Bacteria were cultivated in Luria broth (Fisher Scientific) at 37°C with vigorous shaking (120 rpm) for 12–18 h in a New Brunswick Scientific shaker incubator 126 (Eppendorf Canada, Mississauga, Ontario). Cells were harvested by centrifugation for 5 min at 525 x g and then resuspended in RPMI media. The concentration of the bacteria was determined by measuring the optical density of the suspension at 600 nm; where OD_600_ = 0.2 in the exponential growth phase.

### Infection of human PBMCs for cell aggregate formation

Cryovials containing frozen PBMCs from liquid nitrogen were transferred to a 37°C water bath. The cells were quickly thawed by holding the cryovials on the surface of the water bath with an occasional gentle flick. Immediately the cells were transferred into a 15 ml Falcon^TM^ tube, with room temperature RPMI growth medium. Cells were pelleted at 525 x g for 5 minutes. Cell number and viability were determined by 0.4% Trypan Blue exclusion assay. The final concentrations of the cells were adjusted to 10 x 10^6^/ml at room temperature.

The extracellular matrix (ECM) used to infect the PBMCs was prepared as described by Kapoor et al. [17]. Briefly, ECM was prepared by mixing 0.95 ml Purecol^®^ collagen solution (Cedarlane) with 50 μl 10x Dulbecco’s phosphate buffered saline (DPBS) (Sigma Aldrich), 4 μl fibronectin (Sigma Aldrich) and 10 μl 1 N NaOH (Sigma Aldrich) per ml of matrix solution and kept on ice (pH 7.0). Then, 5 × 10^5^ PBMCs at room temperature were mixed with 50 μl ECM per well of a 96 well plate. For *Mtb*-infected samples, 5 x 10^6^ *Mtb* H37Ra were added to the PBMCs-ECM mixture for a multiplicity of infection (MOI) of 0.1 (1:10). The MOI relative to the monocyte population was 10 (10:1) based on an estimate of 10% PBMCs are monocytes. *Mtb* infects monocytes not lymphocytes [27] and infection stimulates monocyte differentiation to macrophages [28]. Uninfected control wells were either left untreated or treated with Concanavalin A (ConA) (Sigma Aldrich) at 1 μg/ml to account for any T cell proliferation. For non-*Mtb* infection controls, PBMCs were infected with *S*. *aureus* or *E*. *coli* at MOI of 0.05 (1:20) since Gram-positive and Gram-negative bacteria should not form cell aggregates. All infected and uninfected samples were incubated at 37°C for 45 min, prior to overlaying with RPMI medium containing 20% human serum (Cedarlane) the samples were then incubated at 37°C with 5% CO_2_ for 8 days (Forma Scientific CO_2_ Water Jacketed Incubator, ThermoFisher Scientific).

### Definition of a host immune cell aggregate structure

Host immune cell aggregates were defined as dense opaque structures that increase in size of 100 μm by day 8 of incubation when viewed under a light microscope at 10x magnification. To confirm aggregates were actual biological phenomena, the following criteria were required within the infection well: 1) evidence of intracellular infection with *Mtb* H37Ra are determined by transmission electron microscope (TEM) from preliminary experiments and, 2) recruitment of T cells and macrophages were differentiated from monocytes upon infection with bacteria into cell aggregates and confirmed by immunofluorescence staining (see above). TEM studies indicated that *Mtb* H37Ra could be internalized inside macrophages for up to 8 days post-infection, after which we observed extensively damaged and lysed host cells (S1 Fig). Therefore, we used day 8 as the final endpoint in our studies and observed the aggregation of T cells and macrophages. *S*. *aureus* and *E*. *coli* served as negative controls.

### Screening for host immune cell aggregate structures using light microscopy

For detection of a granuloma-like structure from days 0 to day 8 of infection, samples were visualized under the Zeiss Axio Scope A1 light microscope (Carl Zeiss Canada Ltd, Toronto). Images were acquired using a Zeiss Axiocam camera (Carl Zeiss Canada Ltd) at 100x magnification.

### Preparation of histological sections of host cell aggregates for staining

On day 8 of incubation, medium was removed from the wells, and replaced with 4% paraformaldehyde (Sigma Aldrich) in which the samples were left overnight. ECM was carefully removed from the wells, samples were paraffin-embedded and sectioned at a histology core lab Alberta Diabetes Institute (University of Alberta). Sections of samples were deparaffinized by immersing the sample sections in three repetitions of xylene for 5 minutes each, followed two washes of 100% ethanol for 10 minutes each preparations, two washes of 95% ethanol for 10 minutes each and three washes of 70% ethanol for 10 minutes each. The sections were then washed twice in double distilled water (ddH_2_O) for 5 minutes each. Deparaffinized sections of samples were microwaved in 0.1 M sodium citrate buffer for 10 min at 98°C, cooled, and blocked with 5% FBS in PBS for 30 min.

Deparaffinized sample sections were incubated with mouse anti-human CD3 antibody (T cell marker) conjugated to FITC and mouse anti-human CD14 antibody (macrophage marker) conjugated to Texas red for 1 hour at room temperature. Sections of samples were washed 3 times with 1% FBS in PBS, and incubated with 4',6-diamidino-2-phenylindole **(**DAPI) stain (Sigma Aldrich) for 10 minutes at room temperature. The sections were again washed 3 times with ddH_2_0, mounted with 50% glycerol in ddH_2_0 (Sigma Aldrich) and viewed using Zeiss Axio Scope A1 fluorescent microscope. Images were acquired with a Zeiss Axiocam camera and Axion Vision software using the Texas red filter setting (excitation wavelength, 515–560 nm; emission wavelength > 590 nm). FITC filter setting (excitation wavelength, 450–500 nm; emission wavelength, > 528 nm), and the DAPI filter setting (excitation wavelength, 600–625 nm; emission wavelength, > 568 nm) (Carl Zeiss).

### Determination of *Mtb* H37Ra colony forming units in infected PBMCs

To measure the bacterial number, on day 0, 3, 5 and 8 post *Mtb* H37Ra infection, culture supernatants were removed from triplicate wells per condition, pooled and stored at -70°C for later measurements of cytokines. The ECM was digested by adding 50 μl of 0.1% collagenase solution (Sigma-Aldrich) to each sample well followed by incubation at 37°C with 5% CO_2_ for 3 h. Infected cells were then lysed in 0.1% Triton X-100 (Sigma Aldrich, Oakville, Ontario). Ten-fold serial dilutions of lysed cells were prepared in PBS and plated on Middlebrook 7H11 Selective Agar (BD Biosciences, Mississauga, Ontario) and incubated at 37°C with 5% CO_2_ for 21–28 days. The number of CFU for each incubation day were divided by the product of the dilution and the plated volume, averaged, and reported as CFUs/ml.

### Cytokine concentrations in cell culture supernatants

Cell culture supernatants were harvested on days 0, 3, 5 and 8 from triplicate wells per condition, pooled and stored at -70°C until use. Supernatants were subsequently thawed on ice for cytokines IL-10, IL-17, IL-4, IL-6, IFN-γ and TNF-α by enzyme-linked immunosorbant assay (ELISA) using commercially available kits as per the manufacturer’s recommendations (BioLegened, San Diego, California). To determine the precise concentrations, dilutions of the supernatants were tested in triplicate. The lower detection limits of the assays were 3.9 pg/ml for IL-10, IL-17 and IL-4; 7.8 pg/ml for IFN-γ; and 31.25 pg/ml for TNF-α and IL-6. Absorbance was measured at 450 nm using an EnVision multilabel reader 2104 (PerkinElmer, Guelph, Ontario). Cytokine concentrations in the test samples were calculated based upon standard curves generated with known concentrations of recombinant human cytokines.

### Statistical analyses

All experiments were performed in triplicate for each donor. Data were analyzed using SPSS software version 13.0 (IBM Analytics, Armonk, New York). The normal distribution of the data was tested by the Shapiro–Wilks test. All values were reported as mean ± standard deviation. Statistically significant differences among the groups were determined using two-way ANOVA, followed by a Bonferroni post hoc test. Values were considered significantly different when p<0.05.

## Results

### Donor histories

A total of 10 blood donor volunteers were recruited based on their answers to the study questionnaire. Five donors declared a prior BCG vaccination history, while five donors declared no BCG vaccination history. BCG+ donors (n = 5) were from India, China, and South America where BCG vaccination is typically administered at the time of birth. All donors who declared a BCG vaccination also had an identifiable BCG scar. One BCG+ donor also had a positive TST history. All BCG- donors (n = 5) were born in Canada where routine administration of BCG vaccine was stopped in the 1970’s and is currently not available. Two BCG- donors declared a negative TST history. Thus, only three donors declared a TST history.

### *Mtb* H37Ra infection induced formation of large immune cell aggregates

**Fig 2:**
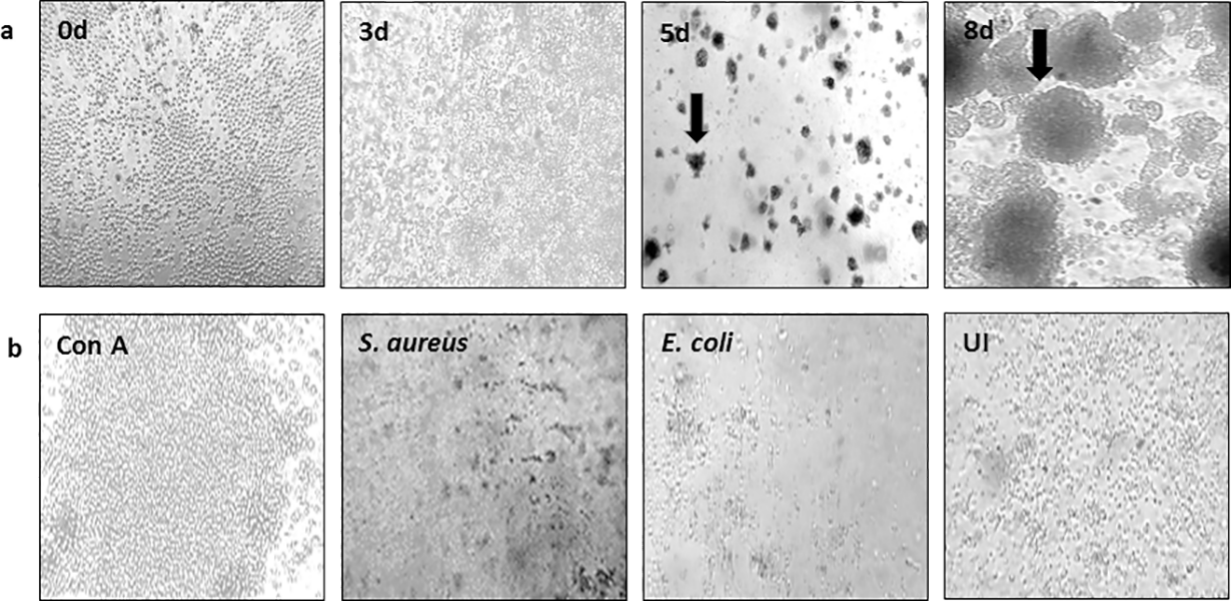
Infection of human PBMCs with *Mtb* H37Ra resulted in formation of cell aggregates. Microscopic examination of (a) human PBMCs infected with *Mtb* H37Ra at culture days 0, 3, 5 and 8, and (b) four control conditions after 8 days culture: uninfected PBMCs treated with ConA, PBMCs infected with *S*. *aureus* or *E*.*coli*, and uninfected (UI) cells. Arrows indicate the location of a representative cellular aggregate. One representative data set is shown out of 10 samples. Experiments were performed three times in triplicate per condition per donor.

Infection of PBMCs with *Mtb* H37Ra resulted in the formation of cellular aggregates at day 3 post-infection (Fig 2A) compared with day 0. However, these cellular aggregates were initially small in size (less than 50 μm in diameter), and became larger as incubation progressed to 5 and 8 days post-infection (Fig 2A). The aggregates grew more compact in appearance, forming sphere-like structures of approximately 100 μm in diameter on average. Control PBMCs obtained from the same donors that were not infected with *Mtb*, or cultured in the presence of ConA, *E*. *coli* or *S*. *aureus* did not form large sphere-like cell aggregates (Fig 2B), indicating that this type of aggregate formation was specific to infection with *Mtb* H37Ra.

### Cell aggregates were composed of T cells and macrophages

**Fig 3:**
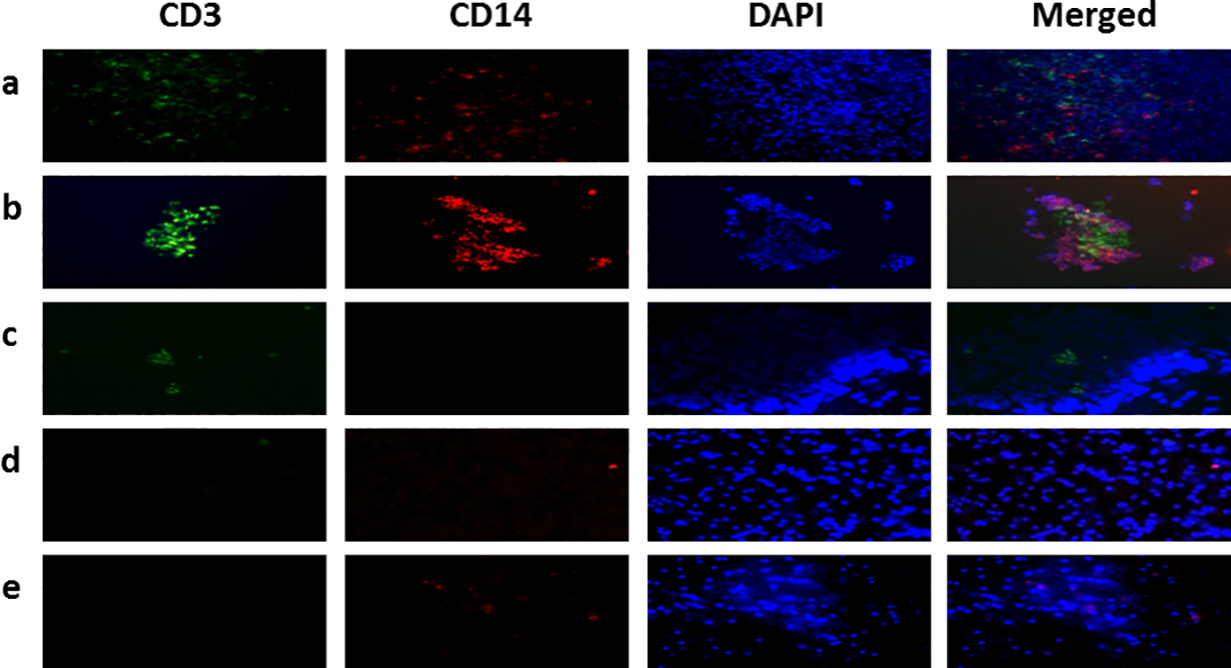
Co-localization of CD3 and CD14 in cell aggregates. Fluorescent stained samples of 8 day cultures in ECM with: (a) uninfected PBMCs (no bacteria control), (b) PBMCs infected with *Mtb* H37Ra, (c) PBMCs treated with ConA (T cell stimulation control), (d) PBMCs infected with *E*. *coli* (Gram-negative bacteria control), (e) PBMCs infected with *S*. *aureus* (Gram-positive bacteria control). CD3 (T cell marker, green), CD14 (macrophage marker, red) and nucleus (blue). One representative data set is shown for each of the 10 PBMC donors.

To identify the cellular components of any cell aggregates, immunostained samples were prepared using fluorescent markers for CD3 (T cell marker), CD14 (macrophage marker), and nuclei were stained with DAPI. T cells and macrophages were observed in both uninfected (Fig 3A) and *Mtb*-infected PBMCs (Fig 3B). However, cell aggregates of T cells and macrophages were only observed in the *Mtb*-infected PBMCs (Fig 3B). The following experimental conditions did not produce cell aggregates: PBMCs treated with ConA (Fig 3C), PBMCs infected with *E*. *coli* (Fig 3D), and PBMCs infected with *S*. *aureus* (Fig 3E).

### *Mtb* H37Ra load in infected PBMCs increased over time

**Fig 4:**
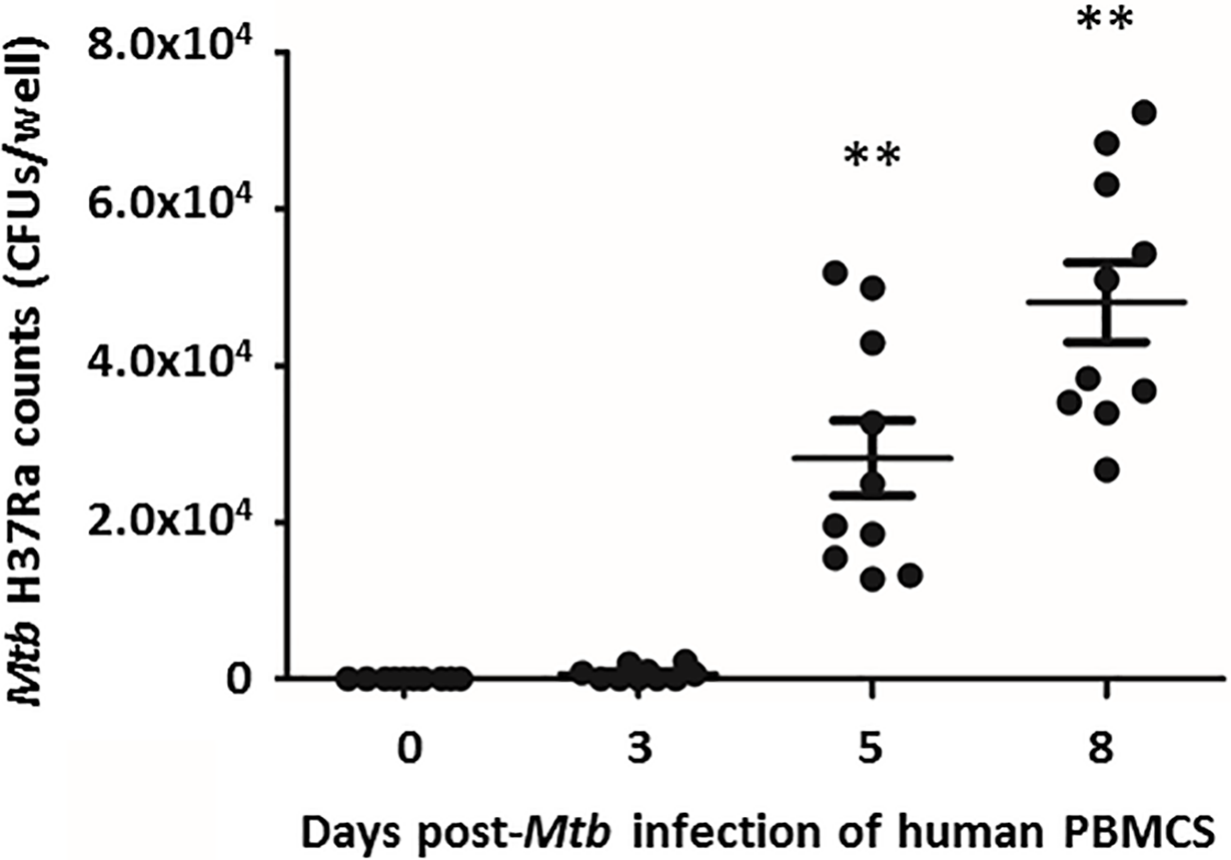
Quantification of *Mtb* H37Ra post-infection on days 0, 3, 5 and 8. Bacterial counts on days 0, 3, 5 and 8 post-infection. Data were plotted as mean ± SD for 10 donors. Experiments were performed in triplicate per condition per donor. **p<0.01 compared to day 0 post-infection.

The number of mycobacterial colony forming units (CFUs) / well were quantified at days 0, 3, 5 and 8 post-infection. Mycobacterial growth between days 0 and 3 post-infection was not significantly different (p = 0.965). However, at days 5 and 8, the number of CFUs/well were significantly increased (p = 0.009) compared to day 0 post-infection (Fig 4).

### Changes in PBMC populations at day 8 post-*Mtb* infection

To evaluate the contribution of naive versus memory cells in response to *Mtb* infection, cell populations were characterized at day 8 post-infection. A comparison of PBMCs stimulated with ConA to unstimulated PBMCs (controls) was performed to validate that immune responses could be measured in PBMCs following collagenase digestion of ECM. As expected, significant differences were observed in the percentage of total Th cells (Table 1) and the percentage of total Tc cells (Table 2) in infected versus uninfected cells. The percentages of activated naïve Th cells CD3^+^CD4^+^CD45RA^+^CCR7^+^CD38^+^HLADR^+^ and activated effector memory Th cells CD3^+^CD4^+^CD45RA^-^CCR7^-^CD38^+^HLADR^+^ were significantly higher (p = 0.032) in infected cells (18.2±0.3) compared to uninfected cells (1.2±3.3) (Table 1). For Tc cells, the percentage of activated central memory cells CD3^+^CD8^+^CD45RA^-^CCR7^+^CD38^+^HLADR^+^ was significantly increased (p = 0.042) in infected (15.3±1.2) versus uninfected cells (3.3±0.4) (Table 2).

**Table 1:** CD4^+^ T cell populations in PBMCs at 8 days post-infection with *Mtb* H37Ra.

Th Cell Populations (Total, Subsets)	Th Cell Phenotype		Percentage of Th Cells Mean ± SD	
			Uninfected (n = 10)	Infected (n = 10)
Total Th cells ^a^	CD3^+^CD4^+^	28.7±4.2	44.2±2.4*
Naïve	CD3^+^CD4^+^*CD45RA*^+^CCR7^+^	16.4±2.3	11.5±1.4
Activated	CD3^+^CD4^+^CD45RA^+^CCR7^+^CD38^+^HLA DR^+^	1.2±3.3	18.2±0.3*
Central Memory	CD3^+^CD4^+^CD45RA^-^CCR7^+^	6.9±3.2	4.5±0.3
Activated	CD3^+^CD4^+^CD45RA^-^CCR7^+^CD38^+^HLA DR^+^	2.7±0.4	1.5±0.5
Effector	CD3^+^CD4^+^CD45RA^+^CCR7^-^	11.2±0.3	16.2±4.7
Activated	CD3^+^CD4^+^CD45RA^+^CCR7^-^CD38^+^HLA DR^+^	13.6±2.2	14.2±1.7
Effector Memory	CD3^+^CD4^+^CD45RA^-^CCR7^-^	32.4±3.2	56.0±5.2
Activated	CD3^+^CD4^+^CD45RA^-^CCR7^-^CD38^+^HLA DR^+^	3.6±2.2	14.2±1.7*

^a^Percent of total lymphocyte population, Uninfected PBMCs, *Mtb*-infected PBMCs

* *p* <0.05, Infected compared to Uninfected

**Table 2:** CD8^+^ T cell populations in PBMCs at 8 days post-infection with *Mtb* H37Ra.

Tc Cell Populations (Total, Subsets)	Tc Cell Phenotype		Percentage of Tc Cells Mean ± SD	
			Uninfected (n = 10)	Infected (n = 10)
Total Tc cells ^a^	CD3^+^CD8^+^	9.7±7.0	25.8±3.5*
Naïve	CD3^+^CD8^+^CD45RA^+^CCR7^+^	3.5±0.5	5.6±1.2
Activated	CD3^+^CD8^+^CD45RA^+^CCR7^+^CD38^+^HLA DR^+^	19.6±1.9	23.7±2.6
Central Memory	CD3^+^CD8^+^CD45RA^-^CCR7^+^	13.1±2.4	10.7±1.5
Activated	CD3^+^CD8^+^CD45RA^-^CCR7^+^CD38^+^HLA DR^+^	.23.3±0.4	15.3±1.2*
Effector	CD3^+^CD8^+^CD45RA^+^CCR7^-^	6.5±1.6	17.6±3.5
Activated	CD3^+^CD8^+^CD45RA^+^CCR7^-^CD38^+^HLA DR^+^	3.6±0.5	6.4±1.0
Effector Memory	CD3^+^CD8^+^CD45RA^-^CCR7^-^	12.9±1.2	15.6±3.2

^a^Percent of total lymphocyte population, Uninfected PBMCs, *Mtb*-infected PBMCs

* *p* <0.05, Infected compared to Uninfected

The percentage of CD3^+^CD4^+^CXCR3^+^CCR6^-^ Th1 cells (43.5±3.1) was significantly greater (p = 0.024) in infected versus uninfected (22.8±2.8) cells. The percentage of CD3^+^CD4^+^CD25^+^ activated T cells was significantly greater (p = 0.026) in the infected (7.2±1.5) compared to the uninfected (3.6±2.1) cells, but no significant difference was observed in the percentage of CD3^+^CD4^+^CD25^+^CD127^+^CCR4^+^ T reg cells in infected (6.2±1.5) and uninfected (5.9±1.2) groups.

### *Mtb* H37Ra infection increased transient production of cytokines on day 3

**Fig 5:**
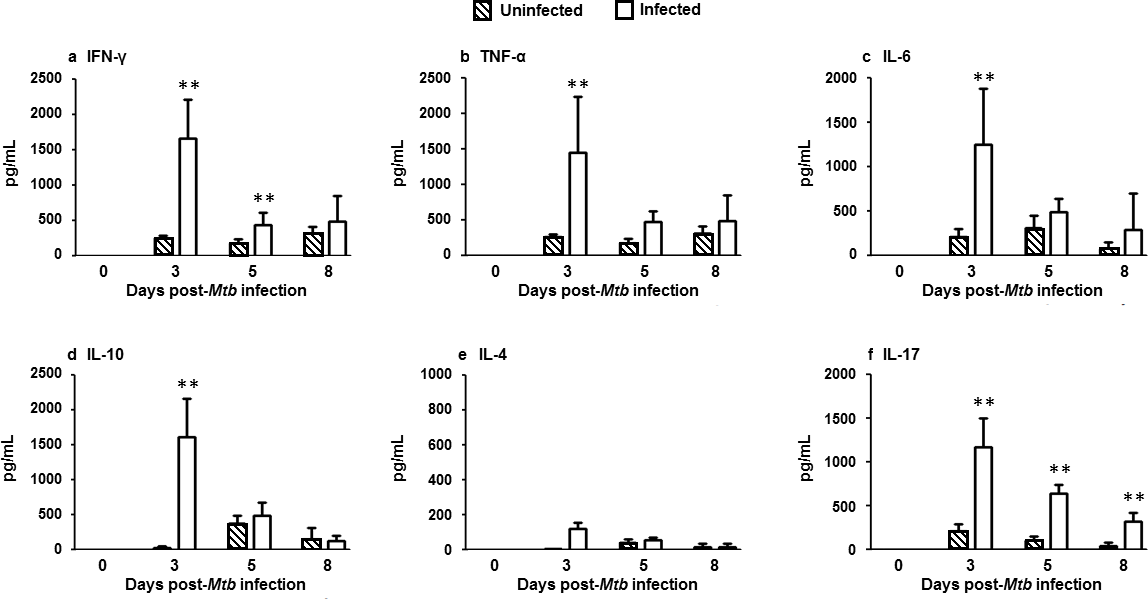
Cytokine levels in cell culture supernatants of PBMCs ± *Mtb* H37Ra infection on days 0, 3, 5 and 8. Cytokine concentrations in cell culture supernatants were determined by ELISA for PBMCs infected with *Mtb* H37Ra, (open bars) uninfected PBMCs (hash bars). Data were plotted as mean ± SD and represent 10 samples per group. Experiments were performed in triplicate per condition per donor. **p<0.01 infected compared to uninfected cells.

The concentrations of cytokines in cell culture supernatants were measured on days 0, 3, 5 and 8 post-infection. The mean concentrations of Th1 cytokine IFN-γ was found to be significantly higher at day 3 (1659±553 pg/ml, p = 0.007) and day 5 (434±182 pg/ml, p = 0.009) post-infection compared to the respective uninfected group at day 3 (246±48 pg/ml) and day 5 (180±66 pg/ml) (Fig 5A). The mean concentrations of other Th1 cytokines were significantly increased only at day 3 in the infected group for TNF-α (1457±637 pg/ml, p = 0.008) (Fig 5B) and IL-6 (1254±626 pg/ml, p = 0.009) (Fig 5C) compared to the mean values observed for the respective uninfected group for TNF-α (246±48 pg/ml) and IL-6 (210±96 pg/ml).

The mean production level of Th2 cytokine IL-10 was also significantly higher (p = 0.008) in the infected group on day 3 (1612±555 pg/ml) compared to the uninfected group (28±19 pg/ml) (Fig 5D). In contrast, the concentration of IL-4 did not vary between infected and uninfected groups (Fig 5E). The production of T cell-derived pro-inflammatory cytokine, IL-17 was significantly higher (p = 0.007) in infected cells on day 3 (1170±333 pg/ml) compared to uninfected cells (216±77 pg/ml), and although infected cell levels fell over time, they remained significantly elevated throughout day eight of infection (Fig 5F).

### Immune responses to *Mtb* H37Ra of PBMCs from BCG vaccinated and BCG unvaccinated donors differ

**Fig 6:**
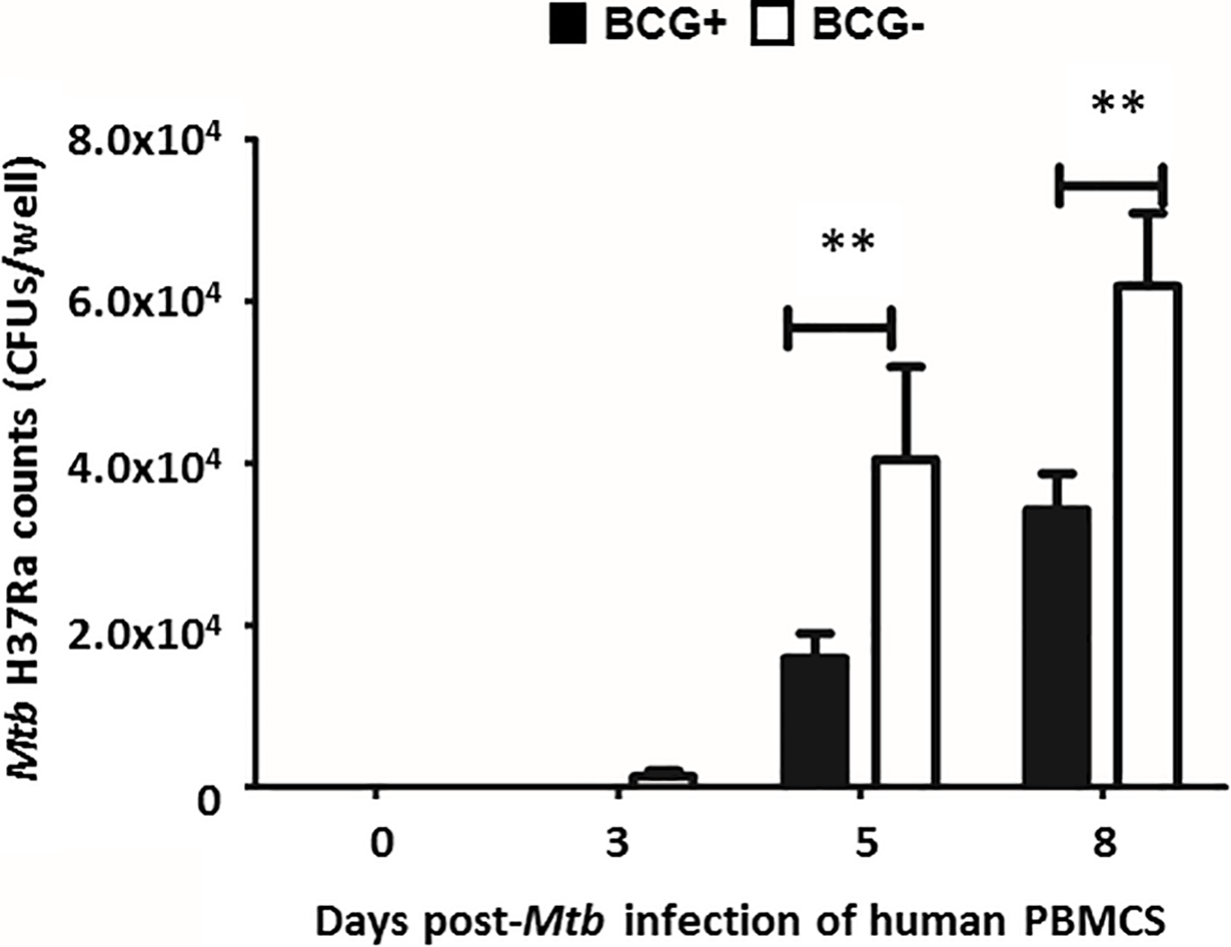
Quantification of *Mtb* H37Ra post-infection in PBMCs from BCG+ and BCG- donors on days 0, 3, 5 and 8. Bacterial counts in PBMCs from BCG+ (solid bars) and BCG- (open bars) donors at days 0, 3, 5 and 8 post-infection with *Mtb* H37Ra. Data were plotted as mean ± SD of 5 samples per group. Experiments were performed in triplicate per condition per donor. **p<0.01, PBMCs from BCG+ donors compared to PBMCs from BCG- donors.

The impact of previous BCG vaccination on immune responses and bacterial growth was assessed by stratifying the 10 samples according to BCG vaccination history. Mycobacterial counts were observed to be significantly lower (p<0.01) in the BCG+ group on days 5 and 8 post-infection compared to the BCG- group, but no significant differences (p = 0.058) were found in bacterial growth between the two groups on days 0 and 3 post-infection (Fig 6).

**Fig 7:**
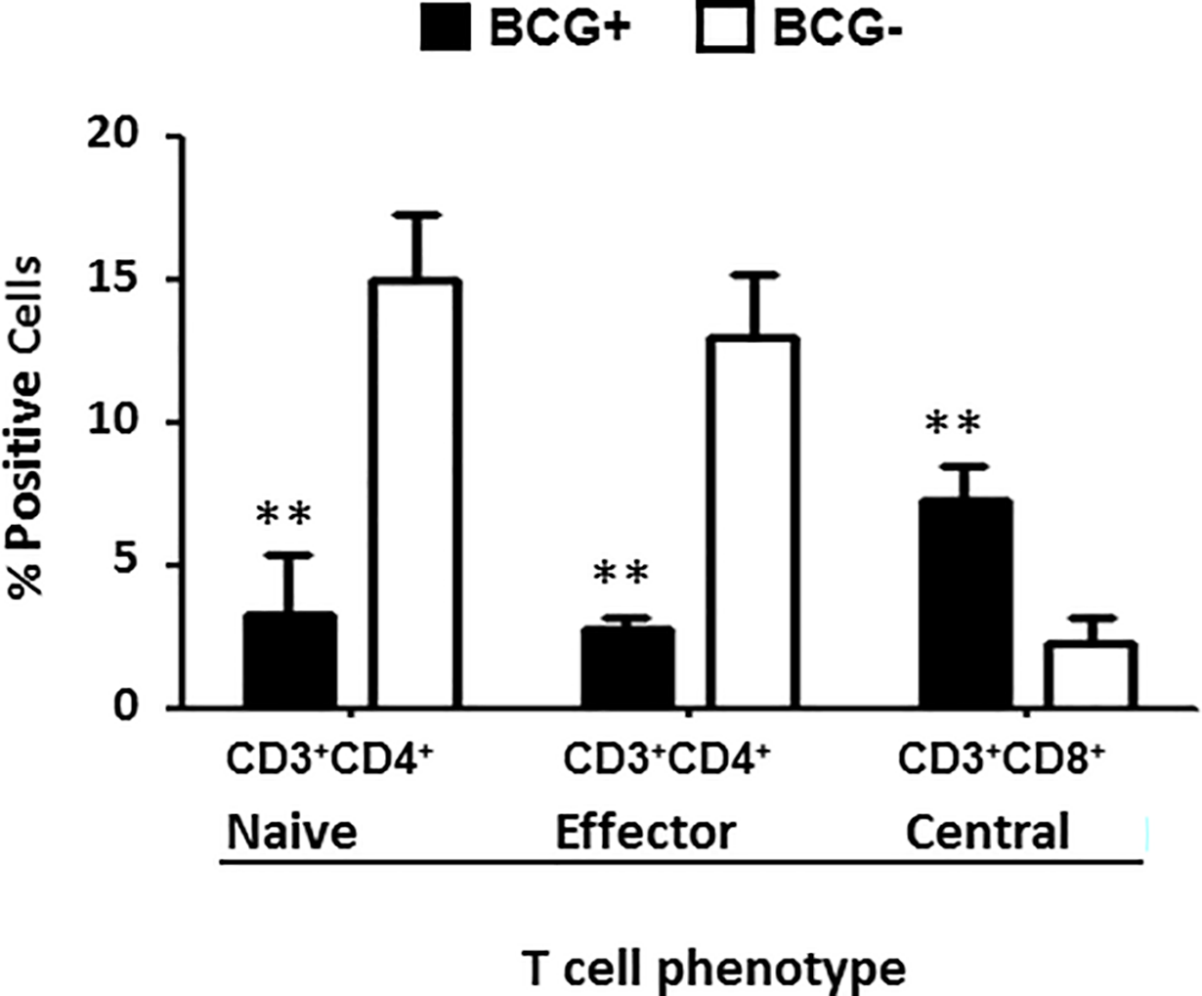
Expression of cell markers on PBMCs from BCG+ and BCG- donors. Activated CD3^+^CD4^+^ and CD3^+^CD8^+^ T cell populations of PBMCs from BCG+ donors (solid bars) and BCG- donors (open bars) infected with *Mtb* H37Ra were determined by flow cytometry at day 8 post-infection: activated naïve CD3^+^CD4^+^ T cells (CD3^+^CD4^+^CD45RA^+^CCR7^+^CD38^+^HLADR^+^), activated effector CD3^+^CD4^+^ T cells (CD3^+^CD4^+^CD45RA^-^CCR7^-^CD38^+^HLADR^+^) and activated central memory CD3^+^CD8^+^ T cells (CD3^+^CD8^+^CD45RA^-^CCR7^+^CD38^+^HLADR^+^). Data were plotted as mean±SD and represent 5 samples per donor group, experiments were performed in triplicate per condition per donor. *p<0.05, PBMCs from BCG+ donors compared to PBMCs from BCG- donors.

There was a difference however in the percentage of activated T cells between PBMCs infected with *Mtb* H37Ra from BCG+ and BCG- donors at day 8 post infection. The percentage of activated naïve Th cells CD3^+^CD4^+^CD45RA^+^CCR7^+^CD38^+^HLADR^+^ (3.3±1.2) and activated effector memory Th cells CD3^+^CD4^+^CD45RA^-^CCR7^-^CD38^+^HLADR^+^ (2.8±1.1) in infected PBMCs from the BCG+ donors were significantly lower (p = 0.032) compared to the respective BCG- donors (15.1±2.2 and 13.0±1.6) (Fig 7). In contrast, the percentage of activated central memory Tc CD3^+^CD8^+^CD45RA^-^CCR7^+^CD38^+^HLADR^+^ cells was higher (p = 0.045) in infected PBMCs from the BCG+ donors (7.4±1.4) compared to the BCG- donors (2.4±1.3) (Fig 7).

**Fig 8:**
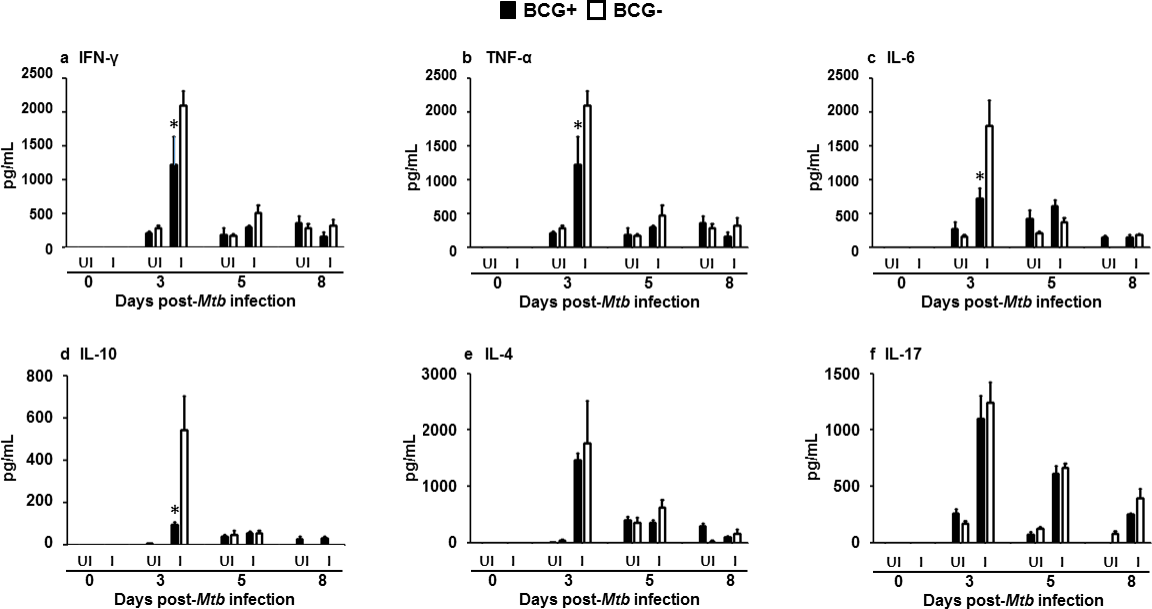
Decreased Th1 and Th2 cytokine production by *Mtb* H37Ra infected PBMCs from BCG vaccinated versus BCG unvaccinated donors. Cytokine concentrations in cell culture supernatants were determined by ELISA at days 0, 3, 5 and 8 for uninfected PBMCs (UI) and PBMCs infected with *Mtb* H37Ra (I) stratified by BCG vaccination status. Data were plotted as mean ± SD and represent 5 samples per donor group. Experiments were performed in triplicate per condition per donor. *p<0.05, compared to BCG+ donors compared to BCG- donor.

The concentrations of cytokines detected in the cell culture supernatants also varied depending upon whether PBMCs were from BCG+ or BCG- donors. On day 3 following infection, the concentrations of Th1 cytokines were lower in cell culture supernatants from the BCG+ donors compared to the BCG- donors (1225±409 *vs* 2095±216 pg/ml) for IFNγ (p = 0.042) (Fig 8A), TNF-α (1169±352 *vs* 2165±226 pg/ml, p = 0.035) (Fig 8B), and IL-6 (718±148 *vs* 1790±377 pg/ml, p = 0.029) (Fig 8C). The concentration of the Th2 cytokine IL-10 was significantly lower (1462±265 *vs* 1761±753 pg/ml, p = 0.031) in cell culture supernatants from the BCG+ group compared to the BCG- group on day 3 post-infection (Fig 8D). At all-time points measured following infection, the concentrations of IL-4 (Fig 8E) and IL-17 (Fig 8F) in cell culture supernatants were not significantly different between BCG+ and BCG- groups.

## Discussion

In this study, *Mtb* growth was the one infection outcome that was most stably confounded by PBMC donor BCG vaccination status at multiple time points over the 8 days infection period. Cytokine expression may be another infection outcome to assess for perturbation by PBMC donor BCG vaccination status; however in our experiments, cytokine expression was generally more transient. In this study we observed a significant increase in the concentration of all three inflammatory cytokines (TNF-α, IFN-γ, IL-6) in the supernatants of PBMCs infected with *Mtb* H37Ra compared to uninfected PBMCs at 3 days post-infection. These increases agree with previous studies where infections of human macrophages with both *Mtb* H37Rv and *Mtb* H37Ra led to TNF-α expression [29]. In addition, TNF-α, IFN-γ, and IL-10 have all been shown to be induced in *Mtb* H37Ra infections of PBMCs from tuberculin-positive donors [30]. Further, IFN-γ production following infection of *Mtb* H37Rv was previously described by similar models involving either the antigen stimulation or infection of PBMCs [17,18,25,31–33]. However, differences in immune responses and mycobacterial growth rates have also been described between models involving infection with *Mtb* H37Rv and *Mtb* H37Ra. For example, H37Ra bacteriostasis and survival in murine macrophages has been previously associated with Th1 cytokine production. In contrast, *Mtb* H37Rv growth within murine macrophages was shown to be associated with expression of Th2 cytokines [34]. Therefore, cytokine expression following *Mtb* infection, may be affected by the eukaryotic cell line as well as the *Mtb* strain used.

In our study, the concentration of the Th2 cytokine IL-10 was higher in supernatants of 3-day infected versus uninfected PBMCs. Increases in Th2 cytokine (e.g. IL-10) concentration following infection may be a result of immune cells attempting to reduce the potentially damaging effects of the Th1 cytokine-mediated inflammatory reactions without impairing the clearance of *Mtb* infection [35]. IL-10 may be produced by a variety of cells identified in the PBMCs we utilized including Th2 (e.g B cells), Treg cells (e.g CD4^+^CD25^+^ Tregs) [36,37] Th1 (e.g CD8^+^ T cells) Th9, Th17 and CD8^+^ T cells [38–41]. Other *in vitro* models have shown expression of both Th1 and Th2 cytokines following challenge with *Mtb* H37Ra. For example, CD4^+^ T cells isolated from broncho alveolar lavages of TB patients stimulated with heat-killed *Mtb* strain H37Ra produced both IFN-γ and IL-10 [42].

The concentration of IL-17 was significantly increased in the culture supernatants of infected versus uninfected PBMCs. Previous studies reported an increase in IL-17 production by T cells isolated from healthy tuberculin test-negative donors in response to *Mtb* antigens [33,43,44]. It is known that IL-17 can be produced by Th17 cells, γδ T cells, and NKT cells [45,46]. IL-17 most likely promotes the development of mature granulomas, since *Mtb*-infected IL-17-knockout mice failed to develop mature granulomas [47]. However, Il-17 may not have a direct impact on mycobacterial load because in an IL-17 deficient mouse model, BCG infection, did not impact bacterial load [48].

In our study, both the percentages of CD4^+^ and CD8^+^ T cells were significantly higher at day 8 in *Mtb-*infected versus uninfected PBMCs. We described an increase in both activated naïve CD4^+^ T cells and activated effector memory CD8^+^ T cells compared to uninfected PBMCs. These patterns of proliferation require further investigation and may be related to the time frame used in our experiments. Cell proliferation patterns may vary when longer time frames are assessed after infection. For example, in a clinical study, *Mtb* infection was associated with CD4 T cell lymphocytopenia without a change in CD8 T cell population [49]. A decrease in CD4^+^ T cells was also demonstrated in an *in vitro* granuloma model using a virulent *Mtb* H37Rv strain [17].

**Fig 9:**
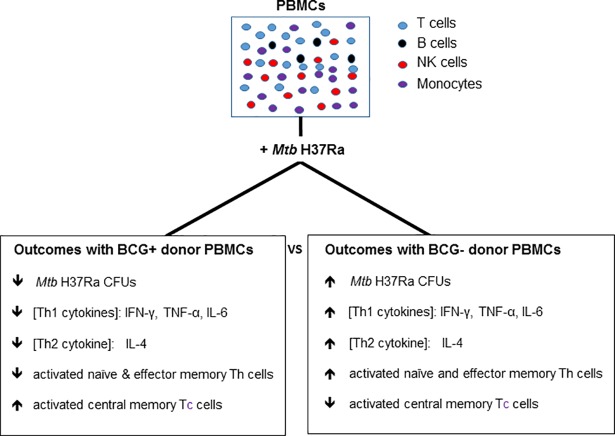
Outcomes of *Mtb* H37Ra growth and cytokine expression in infections of PBMCs from BCG+ compared to BCG- donors. Infections of PBMCs from BCG+ donors were associated with lower *Mtb* H37Ra CFUs, lower concentrations of Th1 and Th2 cytokines, but higher proportions of activated central memory CD8^+^ T cells compared to infections of BCG- donor PBMCs. Closed square brackets ([]) indicate concentration.

In a previous study, bacteriostasis and survival of *Mtb* H37Ra within murine macrophages was associated with induction of Th1 cytokines [34]. Similarly, we observed an association of higher Th1 cytokine concentrations with higher *Mtb* H37Ra CFUs and lower Th1 cytokine concentrations with lower *Mtb* H37Ra CFUs. However, in our experiments, these cytokines and mycobacterial responses are affected by PBMC donor BCG vaccination history. In the present study, we observed lower concentrations of Th1 cytokines (IFN-γ, TNF-α, IL-6) on day 3 post-infection) and lower concentrations of one Th2 cytokine (IL-10) in infections of BCG+ donor PBMCs compared to BCG- donor PBMCs (Fig 9). Similar trends may be seen in the literature with different models of infection. Cattle that were first vaccinated with BCG and then infected with *M*. *bovis* had lower level of IFN-γ, TNF-α, and IL-2 than unvaccinated cattle [50]. However, in our study these changes in Th1 and Th2 cytokines are transient and may not be as effective markers for the assessment of confounding factors as the more stable trends in growth seen with *Mtb* CFUs.

T cell subset populations characterized at day 8 post-infection with *Mtb* H37Ra were associated with a decrease in the percentage of activated naïve and activated effector memory T helper cells in infected PBMCs from BCG+ versus BCG- donors. However, much of what is currently known about T cell subset populations is limited to studies in mouse models [51–54]. While these studies have shown potentially important roles in the host response to *Mtb* infection, evidence of their protective roles remain unclear. We speculate that the higher levels of activated naïve T helper cells in the PBMCs from BCG- donors may reflect an immune response to a primary *Mtb* exposure with significant levels of bacterial replication [55]. It is possible that the increase in activated effector memory T cells (within day 8) in BCG- *vs* BCG+ PBMCs represents a shift from naïve T helper cells in an unvaccinated population [56]. In contrast, we observed an increase in the percentage of activated central memory cytotoxic T cells in PBMCs from BCG+ donors infected with *Mtb* H37Ra compared to BCG-donors at day 8 post-infection. Central memory T cells are thought to provide long-term protection to mycobacterial infection and also generate effector memory and effector cells [57]. A previous experiment by Li et al., identified the proliferation of both functional effector and central memory T cells following BCG infection of PBMCs from BCG- donors [58]. Further, it has been demonstrated that BCG vaccinated cattle that were challenged by *M*. *bovis* produced expanded effector and central memory T cell populations [50]. A recent review identified both central and effector memory T cell populations as being key targets of activation in *Mtb* vaccine studies [59]. In CD4KO mice, it has been demonstrated that protective effector or memory CD8^+^ T cells can be maintained by BCG vaccination without continuous boosting of the vaccine [60].

We acknowledge that the series of experiments presented in this study have some limitations. Host immune responses in human PBMCs were assessed against attenuated *Mtb* H37Ra strain. Since the 1940s, both strains of *Mtb* H37Ra and *Mtb* H37Rv have been widely used for studying the pathogenesis of *Mtb* [61]. Previous work has indicated that the choice of strains (e.g. virulent H37Rv *vs* attenuated H37Ra) can affect the cytokine responses and the growth or survival of mycobacteria in murine macrophage models of infection. In particular, bacteriostasis/survival (instead of death) of *Mtb* H37Ra was associated with induction of Th1 cytokines, while growth of *Mtb* H37Rv was associated with induction of Th2 cytokines [34]. Although use of attenuated strains in infection models may not mirror infection with wild-type virulent strains, use of attenuated strains allow us to assess the impact of potential confounders on experimental models using tools that are outside of a Biosafety Level 3 laboratory [24].

BCG vaccination history was hypothesized to be a potential confounding variable in studies of the early host immune response to *Mtb* infection. In this study, BCG vaccination status was established by donor recall, which may be considered controversial but is commonly used in vaccine effectiveness studies for other pathogens [62]. Misclassification of BCG vaccination status was further reduced by physician evaluation of a declared immunization scar and a review of the BCG vaccination strategies in the country of birth in donors declaring a prior BCG vaccination. All of the BCG+ donors were immigrants from countries where BCG vaccination is commonly administered at the time of birth and this contributed to the limited number of BCG+ participants we were able to recruitfor this study [63]. In contrast, donors who declared no BCG vaccination history, all came from Canada where BCG is not routinely used. Although BCG vaccination does not always result in a scar and scars can wane with time, all BCG+ donors in our study exhibited an identifiable scar [64–66]. We also note that this study focused on host responses early after infection using an attenuated strain of *Mtb*. *Mtb* H37Ra was used in these experiments as it can initiate early host immune responses and can be used in research laboratory settings with limited access to enhanced biosafety facilities. This study did not address possible variations in responses due to infections with wild-type strains of *Mtb* or as infection occurs over a longer time period.

In conclusion, the findings in this study identified a potential confounding effect of prior BCG vaccination history on cytokine profiles and mycobacterial loads in infection models using human donor PBMCs. In particular, mycobacterial loads were clearly impacted by PBMC donor history of BCG vaccination very early following infection (Fig 9). In addition, our preliminary results revealed an impact of BCG vaccination history on T cell populations following PBMC infection with *Mtb* H37Ra, with higher levels of central memory T cells from BCG+ donors compared to BCG- donors. Central memory T cells have been identified to play a key role in the response to *Mtb* infections in mice previously vaccinated with a recombinant BCG strain [67]. However, more work is required to determine the role of these T cells in controlling *Mtb* infection in a human PBMC model of *Mtb* infection. In particular, the present study demonstrates that use of the attenuated strain, *Mtb* H37Ra, allows for the assessment of potential confounding factors to infection models and can still act as a valuable tool to investigate and characterize key elements of the early human host response to *Mtb* infection in a less stringent biosafety environment prior to initiation of experiments with the more pathogenic wild-type strains in Biosafety Level 3 laboratories.

## Supporting information

S1 Table Antibody panels for PBMC immunophenotyping. APC-allophycocyanin; APC-H7-allophycocyanin–cyanine H7; CCR, CC-chemokine receptor; CXCR3, CXC-chemokine receptor 3; DC, dendritic cell; FITC, fluorescein isothiocyanate; NK, natural killer; PE, phycoerythrin; PE-Cy7, phycoerythrin–cyanine 7 tandem; PerCP-Cy5.5, peridinin chlorophyll protein–cyanine 5.5 tandem; Th, T helper; TReg, T regulatory; V421, violet 421; V510, violet 510.
(TIF)

S1 Fig TEM shows lysis of infected macrophages on day 9 post-infection with *Mtb* H37Ra. Representative *Mtb* H37Ra are indicated by a double-headed arrow. The data shown is representative of 3 donors from 8 individual cells observed.
(TIF)
