# Supplementary figures and images for: Characterization of immune responses of human PBMCs infected with Mycobacterium tuberculosis H37Ra: Impact of donor declared BCG vaccination history on immune responses and M. tuberculosis growth
Source: PLoS One. 2018 Sep 11;13(9):e0203822. doi: 10.1371/journal.pone.0203822 (PMC6133369; doi:10.1371/journal.pone.0203822)

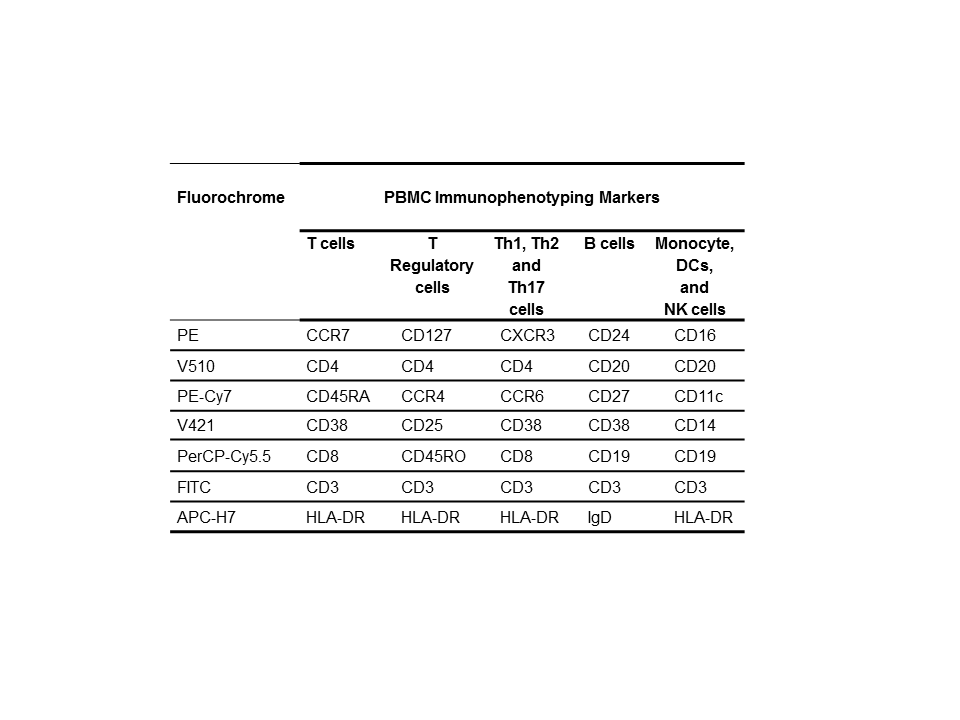

Supplement: S1 Table — APC-allophycocyanin; APC-H7-allophycocyanin–cyanine H7; CCR, CC-chemokine receptor; CXCR3, CXC-chemokine receptor 3; DC, dendritic cell; FITC, fluorescein isothiocyanate; NK, natural killer; PE, phycoerythrin; PE-Cy7, phycoerythrin–cyanine 7 tandem; PerCP-Cy5.5, peridinin chlorophyll protein–cyanine 5.5 tandem; Th, T helper; TReg, T regulatory; V421, violet 421; V510, violet 510. (TIF) [file pone.0203822.s001.tif]

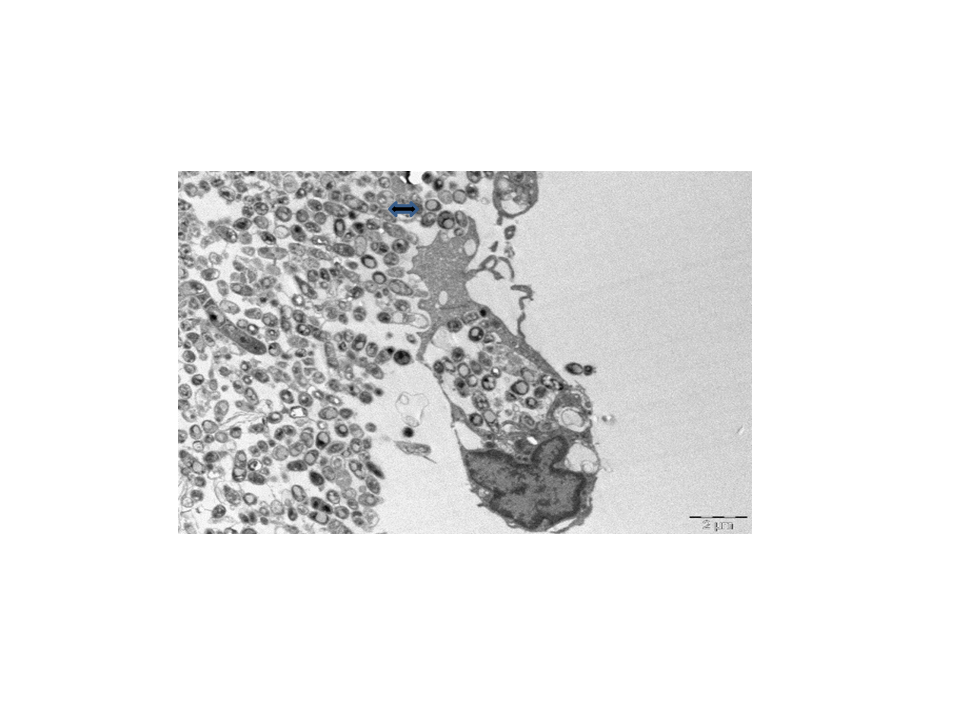

Supplement: S1 Fig — Representative Mtb H37Ra are indicated by a double-headed arrow. The data shown is representative of 3 donors from 8 individual cells observed. (TIF) [file pone.0203822.s002.tif]
